# CAR-Associated Vesicular Transport of an Adenovirus in Motor Neuron Axons

**DOI:** 10.1371/journal.ppat.1000442

**Published:** 2009-05-22

**Authors:** Sara Salinas, Lynsey G. Bilsland, Daniel Henaff, Anne E. Weston, Anne Keriel, Giampietro Schiavo, Eric J. Kremer

**Affiliations:** 1 Molecular NeuroPathobiology Laboratory, Cancer Research UK London Research Institute, London, United Kingdom; 2 Institut de Génétique Moléculaire de Montpellier, CNRS UMR 5535, Montpellier, France; 3 Universités de Montpellier I & II, Montpellier, France; 4 Electron Microscopy Laboratory, Cancer Research UK London Research Institute, London, United Kingdom; University of Michigan Medical School, United States of America

## Abstract

Axonal transport is responsible for the movement of signals and cargo between nerve termini and cell bodies. Pathogens also exploit this pathway to enter and exit the central nervous system. In this study, we characterised the binding, endocytosis and axonal transport of an adenovirus (CAV-2) that preferentially infects neurons. Using biochemical, cell biology, genetic, ultrastructural and live-cell imaging approaches, we show that interaction with the neuronal membrane correlates with coxsackievirus and adenovirus receptor (CAR) surface expression, followed by endocytosis involving clathrin. In axons, long-range CAV-2 motility was bidirectional with a bias for retrograde transport in nonacidic Rab7-positive organelles. Unexpectedly, we found that CAR was associated with CAV-2 vesicles that also transported cargo as functionally distinct as tetanus toxin, neurotrophins, and their receptors. These results suggest that a single axonal transport carrier is capable of transporting functionally distinct cargoes that target different membrane compartments in the soma. We propose that CAV-2 transport is dictated by an innate trafficking of CAR, suggesting an unsuspected function for this adhesion protein during neuronal homeostasis.

## Author Summary

Adenoviruses commonly cause subclinical morbidity in the ocular, respiratory, and gastrointestinal tracts, and less frequently, adenovirus-induced disease can be fatal for newborns and immunocompromised hosts. In addition, adenoviruses can reach the central nervous system (CNS) and cause associated encephalitis and tumours. On the flip side, during the last two decades, adenovirus vectors have become powerful tools to treat and address diseases of the CNS. Despite the fact that axonal transport of adenoviruses was reported more than 15 years ago, nothing was known concerning how adenoviruses access the CNS. The characterization of their interactions with brain cells was therefore long overdue. In this study, we describe the axonal trafficking of an adenovirus that preferentially infects neurons and reaches the CNS through long-range axonal transport. We show that this adenovirus exploits an endogenous vesicular pathway used by the adhesion molecule CAR (coxsackievirus and adenovirus receptor). Our study characterizes this endogenous route of access, which is likely to be crucial to neuronal survival, neurodegenerative diseases, gene transfer vectors, and adenovirus-induced morbidity.

## Introduction

*Adenoviridae* is a family of greater than 150 nonenveloped double-stranded DNA viruses that infect all vertebrate classes. Whilst adenoviruses (Ads) are commonly associated with respiratory, ocular and gastrointestinal tract infections, many serotypes cause clinical manifestations in other tissues, including the central nervous system (CNS) [1]–[4]. Interest in Ad biology has been rekindled by at least two events: Ads have re-emerged as life-threatening pathogens in immunosuppressed hosts and young military recruits [5], and they are currently the most common viral vectors used in clinical gene transfer trials. Importantly, Ad infections can be lethal in immunocompromised patients due to genetic defects (SCID), during haematopoietic stem cell transplants or by pharmacological agents (e.g. during solid organ transplant) [2].

For brain-directed gene transfer, Ad vectors, in particular canine serotype 2 (CAV-2) [6] have unique characteristics. In the CNS of rodents, dogs and primates (including human tissue *ex vivo*), CAV-2 vectors preferentially transduce neurons and undergo efficient axonal transport ([7]; our unpublished data). We previously demonstrated that following interstriatal injections in rodents, CAV-2 was transported to afferent structures such as the contralateral and ipsilateral cortex, substantia nigra, thalamus and basal nuclei of Meynert [7]–[9]. In addition, following injection into the mouse gastrocnemius, CAV-2 preferentially transduced motor neurons of the sacral dorsolombar rachis [7]. CAV-2 vectors also lead to >1 year *in vivo* transgene expression in rodent CNS [8],[9] without accompanying immunosuppression. In addition to their potential in addressing fundamental neurobiological questions [9]–[11], these molecular tools could also be used for treatment of neurodegenerative disorders [12].

Although there are a handful of exceptions, most Ad attachment and trafficking studies have used epithelial-like cells and serotypes from human subgroup B, C and D (e.g. Ad2, 5, 35 and 37). Many human serotypes, as well as CAV-2, bind with high affinity to the coxsackievirus and adenovirus receptor (CAR) [13]–[16], a widely expressed cell adhesion protein involved in tight junction formation in epithelial cells and myocardial cells, and highly expressed in the developing brain [17]–[21]. Many CAR-tropic Ads are endocytosed in clathrin- and Rab5-associated pathways in epithelial cells [22]–[24]. Following receptor-mediated internalisation, subgroups C Ads are thought to undergo a stepwise disassembly, starting with detachment of the fibre from the virus at the cell surface, followed by a passage through early endosomal compartments in which acidification serves as a disassembly trigger [25],[26]. Although the mechanism is poorly understood, intra-endosomal signals likely release vertex proteins, which may lead to protein VI-mediated membrane lysis [27] and escape of the virion into the cytosol [25]. The metastable virions may then be targeted via dynein and microtubule-dependent mechanisms towards the nucleus in some cell types [28]–[30].

In spite of initial reports demonstrating that Ad vectors can be transported retrogradely in neurons *in vivo* [31],[32], little is known concerning their brain cell receptors, the endosomal compartment(s) entered during trafficking or the determinants for their long-range transport. Axonal transport is crucial for neuronal differentiation and homeostasis, which depend on the efficient long-distance delivery (up to 1 meter in humans) of signals and cargoes [33]. This pathway relies mainly on the microtubule-based motors kinesins and cytoplasmic dynein, and their coordination with F-actin-based motors [33],[34]. Alterations in components of the axonal transport machinery are associated with a growing number of neurodegenerative conditions, including Alzheimer's, Parkinson's, Huntington's and motor neuron diseases [33],[35]. In spite of its importance, we are only beginning to understand how the machinery of axonal transport is regulated.

The dual nature of Ads as ubiquitous pathogens and potential gene transfer vectors for the CNS, imposes an in-depth analysis of the molecular mechanisms involved in the virus-neuron interaction. Here, we characterised the binding, internalisation and axonal transport of an Ad that preferentially infects neurons. Our data suggest that the neuronal binding of CAV-2 is CAR-dependent and its internalisation involves clathrin-coated pits and the small GTPase Rab5. In contrast to the established paradigm of Ad trafficking in epithelial cells, long-range CAV-2 transport in axons is mainly vesicular, and depends on the sequential maturation of transported endosomes, which switch from Rab5 to Rab7. We found that CAV-2 axonal motility is bidirectional, with a bias for the retrograde direction. Carriers of CAV-2 also transported tetanus toxin and neurotrophin receptors and surprisingly still contained CAR. We also demonstrated that similarly to whole virions the fibre knob (FK) protein could be found in CAR^+^ organelles. We therefore propose that the intrinsic neuronal properties of CAR are responsible for the efficient trafficking of CAV-2 in neurons. More globally, our data demonstrates that distinct receptor-mediated endocytic events determine the sorting of diverse cargoes to nonacidic vesicles, which are then recruited in a Rab7-dependent manner to the long-range retrograde transport pathway, in a process that allows selected pathogens to reach the CNS.

## Results

### CAV-2 binding correlates with CAR surface expression and uptake involves clathrin-coated pits

**Figure 1:**
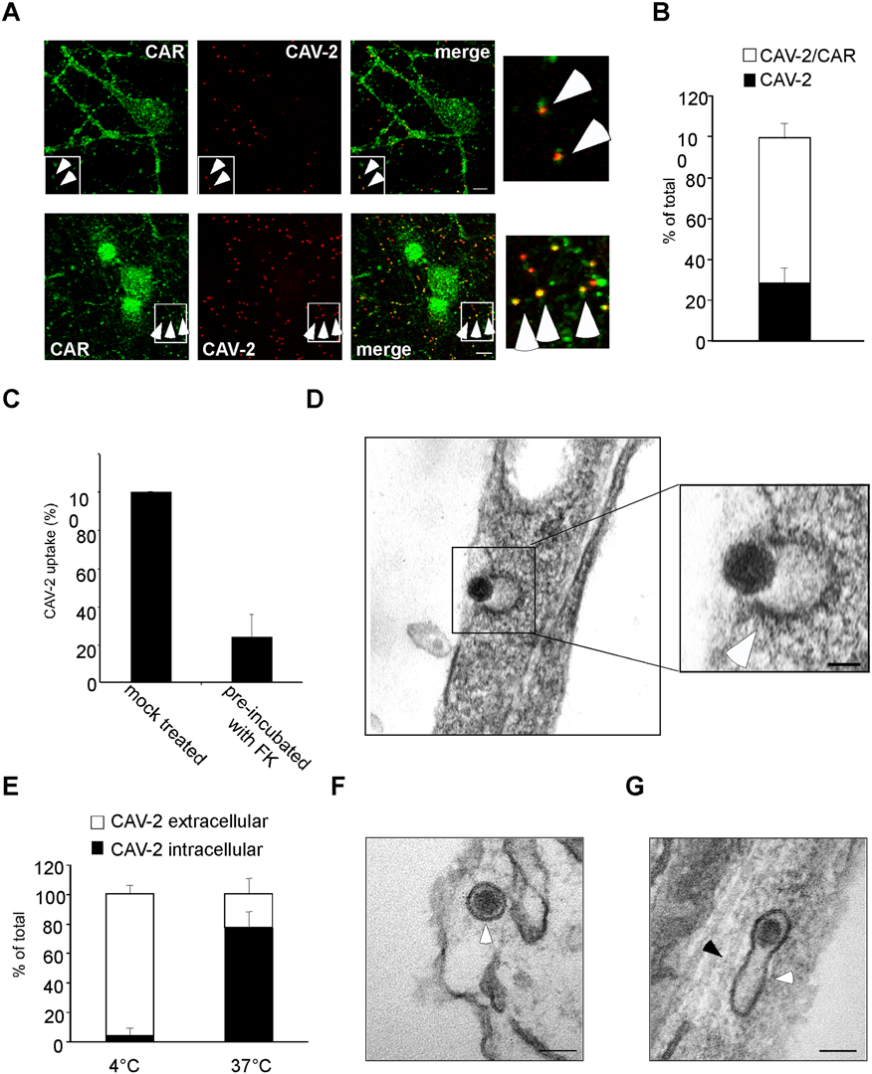
CAV-2 binding and internalisation in MNs. (A) MNs and DRG were incubated with CAV-Cy3 (red) on ice, fixed and stained for total (intracellular and plasma membrane bound) CAR (green). The white rectangle is enlarged to show colocalisation of CAV-2 and CAR on axons. (B) Quantification of the colocalisation between CAV-2 and CAR in MNs (3 independent experiments, 149 virions total). The white bar corresponds to colocalised CAV-Cy3 and CAR (yellow puncta) and the black bar corresponds to CAV-Cy3 alone (red puncta). Error bars (SEM) represent 7.2%. (C) CAV-2 uptake is severely impaired by pre-treatment of MNs with an excess of recombinant FK (see Materials and Methods; 3 independent experiments, 670 (untreated) and 475 (FK) virions in total. Error bar represents 11.9%). (D, F and G) Ultrastructural analyses of CAV-2 entry in MNs. Cells treated with CAV-2 were fixed at different time points and neurites imaged by TEM. (D) At early time-points (1 minute), CAV-2 was found associated preferentially with structures resembling clathrin-coated pits (arrow). (E) MNs were incubated with CAV-Cy3 on ice and either fixed or shifted to 37°C for 45 minutes to allow internalisation. Extracellular virions were revealed by using an anti-Cy3 antibody by IF and quantified (3 independent experiments, 307 (4°C) and 522 (37°C) virions in total. Error bars represent 5.6% (4°C) and 10.7% (37°C)). (F) After 2 minutes of internalisation, CAV-2 was mainly present in vesicular structures (arrow) juxtaposed to the plasma membrane. (G) Endocytic tubule (white arrow) containing CAV-2 nearby microtubule tracks (black arrow). Scale bars: (A) 5 µm; (D) 50 nm; (F, G) 100 nm.

CAV-2 vectors preferential infect neuronal cells *in vivo* and in mixed brain cell cultures, however the binding determinants responsible for this tropism have not been addressed. Although the 150 Ad serotypes can bind numerous co-receptors [36],[37], our previous studies suggested that CAR is the main receptor for CAV-2 [16],[38]. To study the neuronal link between CAR and CAV-2, we incubated Cy3-labelled CAV-2 virions (CAV-Cy3) with primary spinal cord motor neurons (MNs) on ice to allow binding, but prevent internalisation. Cells were then fixed and stained for endogenous CAR. Interestingly, CAR was found in two distinct compartments in MNs. In addition to a plasma membrane localisation seen also in sparse epithelial-like cells copurifying with MNs, CAR was also found in a large intracellular pool (Figure S1A). We found that >70% of CAV-Cy3 colocalised with CAR on neurites in MNs and dorsal root ganglia neurons (DRG) (Figures 1A, B and S1B). Moreover, when MNs were pre-incubated with saturating concentrations of recombinant fibre knob (FK), the adenovirus protein responsible for CAR binding, and then treated with CAV-2, virion uptake was reduced by 76% compared to control (Figure 1C).

We then examined the early steps of CAV-2 entry in MNs by transmission electron microscopy (TEM). At 1 minute post-internalisation, electron dense CAV-2 virions were associated with structures resembling clathrin-coated pits, often present at cell-to-cell contacts (Figures 1D, S1C and data not shown). By indirect immunofluorescence, we also found extensive colocalisation between clathrin heavy chain and CAV-2 (Figure S1D). These results are in good agreement with previous reports showing that in epithelial cells, CAR-tropic Ads undergo clathrin-associated endocytosis, and are consistent with our current understanding of CAV-2 internalisation in these cells [16],[39]. We next assessed CAV-2 internalisation in MNs. To this end, we again incubated MNs with CAV-Cy3 on ice and then replaced the medium with warm medium to induce internalisation. Cultures were incubated at 37°C for 45 minutes, then shifted back to 4°C and incubated with anti-Cy3 antibody to detect surface-bound virions. We found that MNs internalised >75% of CAV-2 under these conditions (Figure 1E).

Upon internalisation in epithelial cells, most CAR-tropic Ads are believed to rapidly exit endosomal compartments to reach the cytoplasm [30] from where the capsid may interact directly or indirectly with cytoplasmic dynein [29], and be transported towards the nucleus. To determine if a similar process was also at the basis of the axonal transport of CAV-2, virions were incubated with MNs at 4°C then shifted to 37°C, fixed at different times and then visualized by TEM. At 2 to 5 minutes post-internalisation, the majority (>90%) of the virions were inside intact endosomal membranes (Figure 1F). Surprisingly, this pattern did not change significantly (∼90%) 30 to 45 minutes post-internalisation, when live imaging of CAV-2 axonal transport was optimal (3 independent experiments, 97 virions in total; see below). At these later time points, membrane-enveloped virions could be detected close to structures morphologically similar to microtubule tracks (Figure 1G, black arrow). Together these results suggest that CAV-2 binds CAR, is endocytosed in clathrin-coated pits and, unexpectedly, remains within endosomal compartments associated with microtubules in MNs.

### Axonal transport of CAV-2 is bidirectional and vesicular in cultured MNs

**Figure 2:**
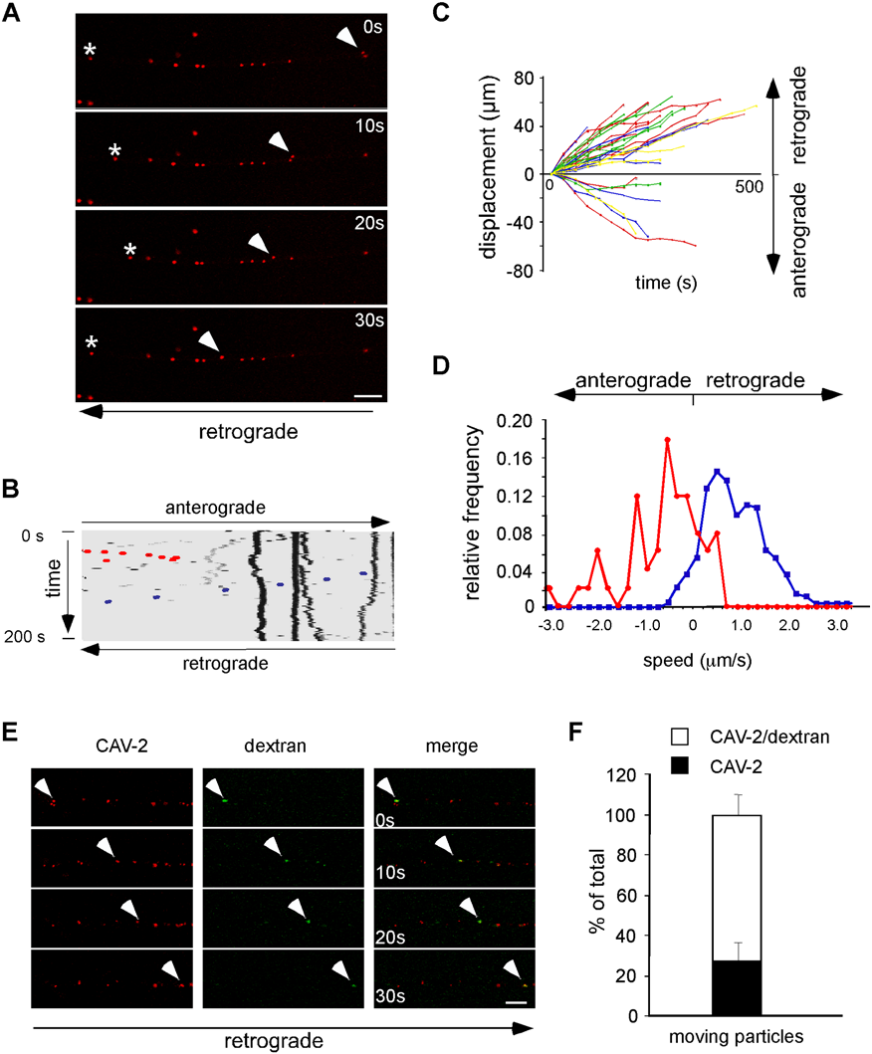
CAV-2 axonal transport in MNs. MNs were incubated with CAV-Cy3 for 45 minutes and imaged by time-lapse confocal microscopy (100 frames; 0.2 frames/s). (A) Individual frames of a movie from a confocal time-series are shown. The cell body is located to the left. Arrowheads show a virion being retrogradely transported, whereas asterisks indicate an anterograde virion stopping and changing to a retrograde direction. See also Video S1. (B) Kymograph of the corresponding movie with a retrograde CAV-2 highlighted in blue. The viral particle labelled by the asterisk in (A) is in red. (C) Displacement graph of 4 independent movies (46 carriers in total). CAV-2 showed a preferential retrograde transport, with only few anterogradely-transported viral particles. (D) Speed distribution profile of CAV-2 in MNs. Blue line: 40 retrograde carriers, 398 single movements. Red line: 6 anterograde carriers, 50 single movements (3 independent experiments). (E) Transported endocytic structures containing CAV-2 were revealed by AlexaFluor647-labelled dextran (false coloured in green) and quantified in (F) (3 independent experiments, 73 carriers in total. Error bars represent 9.6%). Scale bars: 5 µm.

The above results prompted us to characterise the motility of intracellular CAV-2 using established vesicular transport markers by live cell imaging. Initially, we incubated CAV-Cy3 with primary MNs, and axons were then imaged by confocal microscopy. Using this approach, we detected bidirectional transport of CAV-2 (Figure 2A and B, Video S1). Whilst the majority (87%) of motile virions were transported towards the soma, some (13%) showed anterograde movement (Figure 2C, lower quadrant). In addition, some single virions changed direction during imaging (Figure 2A and B, asterisk and red dotted line), suggesting that either CAV-2 structures associates with molecular motors of different polarity or that dynein-dependent bidirectional transport [40] influences its kinetic properties. Bidirectional CAV-2 transport, with a preference for retrograde motility, was also found in cultures of embryonic DRG (data not shown), suggesting a similar mechanism in sensory neurons. The kinetics of transport were analysed by determining the speed distribution profile of CAV-2 in MNs (Figure 2D). CAV-2 retrograde transport appeared to be bimodal with peaks at 0.60 and 1.30 µm/s (Figure 2D, blue line), which is consistent with fast retrograde transport [41]. In contrast, the anterograde transport profile was more discontinuous (Figure 2D, red line).

While characterising CAV-2 transport kinetics, we noticed a delay in the onset of long-range axonal transport. Although our results suggested that CAV-2 is rapidly internalised (<5 min; Figure 1F), we detected primarily oscillatory movements at early times post-internalisation (Figure S2, top panel). Only after 25 minutes were we able to detect long-range movements (Figure S2, middle panel), with robust vectorial transport beginning after ∼30 minutes (Figure S2, middle and lower panels).

In contrast to the efficient escape from endosomes by CAR-tropic Ads, our TEM data showed that the majority of CAV-2 remained trapped in vesicles when axonal transport is most efficient. To directly address the possibility that CAV-2 axonal transport is mediated by a membrane compartment, we co-incubated MNs with CAV-Cy3 and AlexaFluor647-dextran, which is a fluid phase marker used to identify endocytic organelles. Consistent with our TEM observations, we found the majority (∼75%) of virions were co-transported with dextran (Figure 2E and F). These data suggest that CAV-2 uses a vesicular transport pathway to reach the MN soma.

### Fast axonal transport of CAV-2 occurs in compartments with pHs close to neutral

**Figure 3:**
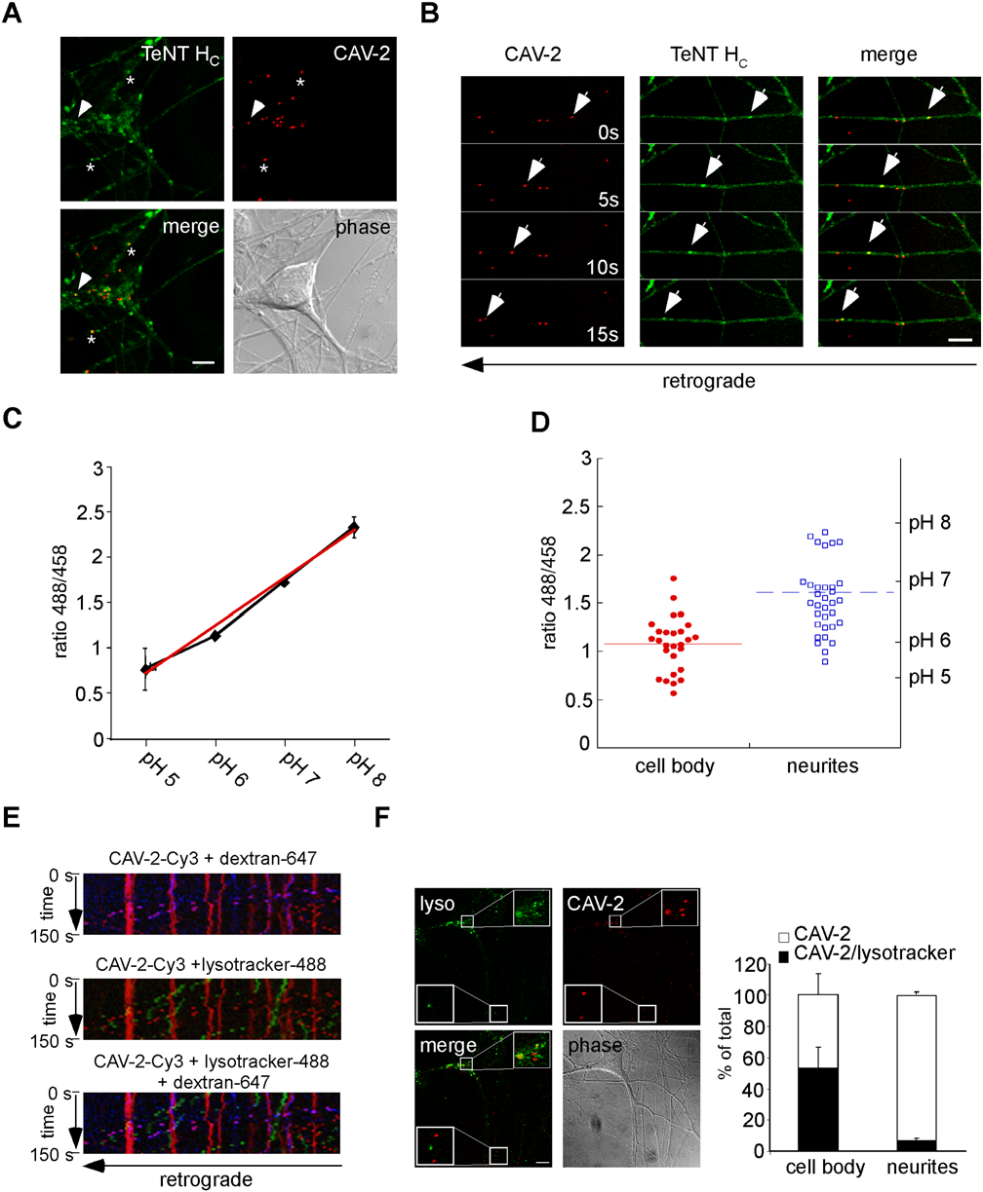
CAV-2 transport in nonacidic retrograde carriers in MN axons. MNs were incubated with CAV-Cy3 and AlexaFluor488-TeNT H_C_ for 45 minutes, fixed and imaged (A) or imaged live (B). (A) CAV-2 and TeNT H_C_ colocalise in neurites (asterisk) and cell bodies (arrowhead) of MNs. (B) Live cell imaging of MNs co-incubated with CAV-Cy3 and AlexaFluor488-TeNT H_C_. Individual frames of a movie are shown. The cell body is located to the left. Arrowheads point to a TeNT H_C_ carrier that is also positive for CAV-2. Greater than 85% of CAV-2 vesicles contained TeNT H_C_ (4 independent experiments, 40 carriers in total). See also Video S2. (C) and (D) MNs were incubated with CAV-CF for 45 minutes and imaged live. (C) pH calibration curve of CAV-2 structures after treating MNs with ionophores and L15 adjusted at different pHs. Red curve is the best fitting curve, with R^2^ = 0.9888. 2 independent experiments. Error bars represent 0.22 (pH 5), 0.0008 (pH 6), 0.026 (pH 7) and 0.11 (pH 8). (D) pHs of somatic versus axonal CAV-CF containing organelles (21 and 33 virions respectively, 2 independent experiments). Each point represents a single CAV-CF-positive structure. The red and blue horizontal lines are the mean pHs of the two pools of organelles. (E) and (F) MNs were incubated with CAV-Cy3, AlexaFluor488-Lysotracker and/or AlexaFluor 647-dextran for 45 minutes and imaged live. (E) Kymographs showing that dextran-containing CAV-2 carriers are nonacidic. (F) Acidic structures containing CAV-2 in the cell body of a MN. Quantitative analyses of CAV-2 structures association with acidic compartments: in the cell body of MNs (4 independent experiments, 163 (cell body) and 211 (neurites) virions in total. Error bars represent 13.6% (CB) and 1.9% (neurites)). Scale bars: (A, B) 5 µm; (F) 10 µm.

The stable association of CAV-2 with the endosomal lumen is inconsistent with the canonical mechanism regulating productive CAR-tropic Ad infections, and may represent a key determinant for efficient axonal transport of CAV-2. Because the exit of Ads from endosomes is triggered by the acidification of their lumen, CAV-2 might enter nonacidic pH compartment(s) allowing its stable sequestration during axonal transport. To test this hypothesis, we assessed the association of CAV-2 with a fragment of tetanus toxin (TeNT H_C_), which is internalised via a clathrin-dependent mechanism coupled to axonal retrograde transport and is sorted to carriers characterised by neutral pH [42],[43]. To this end, we co-incubated MNs with CAV-Cy3 and fluorescently-labelled TeNT H_C_ [41]. In fixed samples, CAV-Cy3 colocalised with TeNT H_C_ in axons and somas (Figure 3A). Furthermore, using live-cell imaging we found that more than 85% of CAV-2 was co-transported with TeNT H_C_ (Figure 3B and Video S2). Our previous work showed that TeNT H_C_ carriers also contain neurotrophins and their receptors [44]. Accordingly, CAV-2 carriers were also positive for the neurotrophin receptor p75^NTR^ (data not shown).

To directly assess the pH of the transport carriers containing CAV-2, MNs were incubated with CAV-2 covalently labelled with carboxyfluorescein (CAV-FC), a probe previously used to measure the pH of endosomes reached by Ads during endocytosis [45]. CAV-FC-infected MNs were incubated with the ionophores nigericin and monensin, exposed to L15 media at different pHs, and the ratio of the emission intensities upon sequential excitation at 458 and 488 nm was determined. Under these conditions, the calibration curve of the pH-dependent fluorescence of CAV-FC was obtained (Figure 3C). We then assayed the pH of CAV-FC-containing structures in neurites compared to cell bodies (Figure 3D). Consistent with the co-transport of CAV-2 with TeNT H_C_, we found that the majority of axonal CAV-FC was within a pH-range of 6 to 7 (Figures 3D). Interestingly, we detected numerous acidic (pH<6) CAV-FC structures in the soma, whereas only very few axonal CAV-FC could be observed at or below pH 6 (Figure 3D).

To test the presence of CAV-2 in nonacidic structures in axons using an alternative approach, MNs were incubated with CAV-Cy3, AlexaFluor647-dextran and Lysotracker-488, a probe that is sequestered in acidic compartments. Consistent with the above results, axonal CAV-2/dextran-positive carriers were Lysotracker-488-negative (Figure 3E). Furthermore, our quantitative analyses of the extent of colocalisation between CAV-2 and lysotracker confirmed the higher association of virions in acidic organelles in cell bodies of MNs versus neurites (Figure 3F). Taken together, these data demonstrate that the majority of CAV-2 is retrogradely transported in axons inside a nonacidic vesicular compartment, which is also used by endogenous ligands, receptors and other pathogens.

### Rab5 to Rab7 endosomal maturation is required for CAV-2 transport

**Figure 4:**
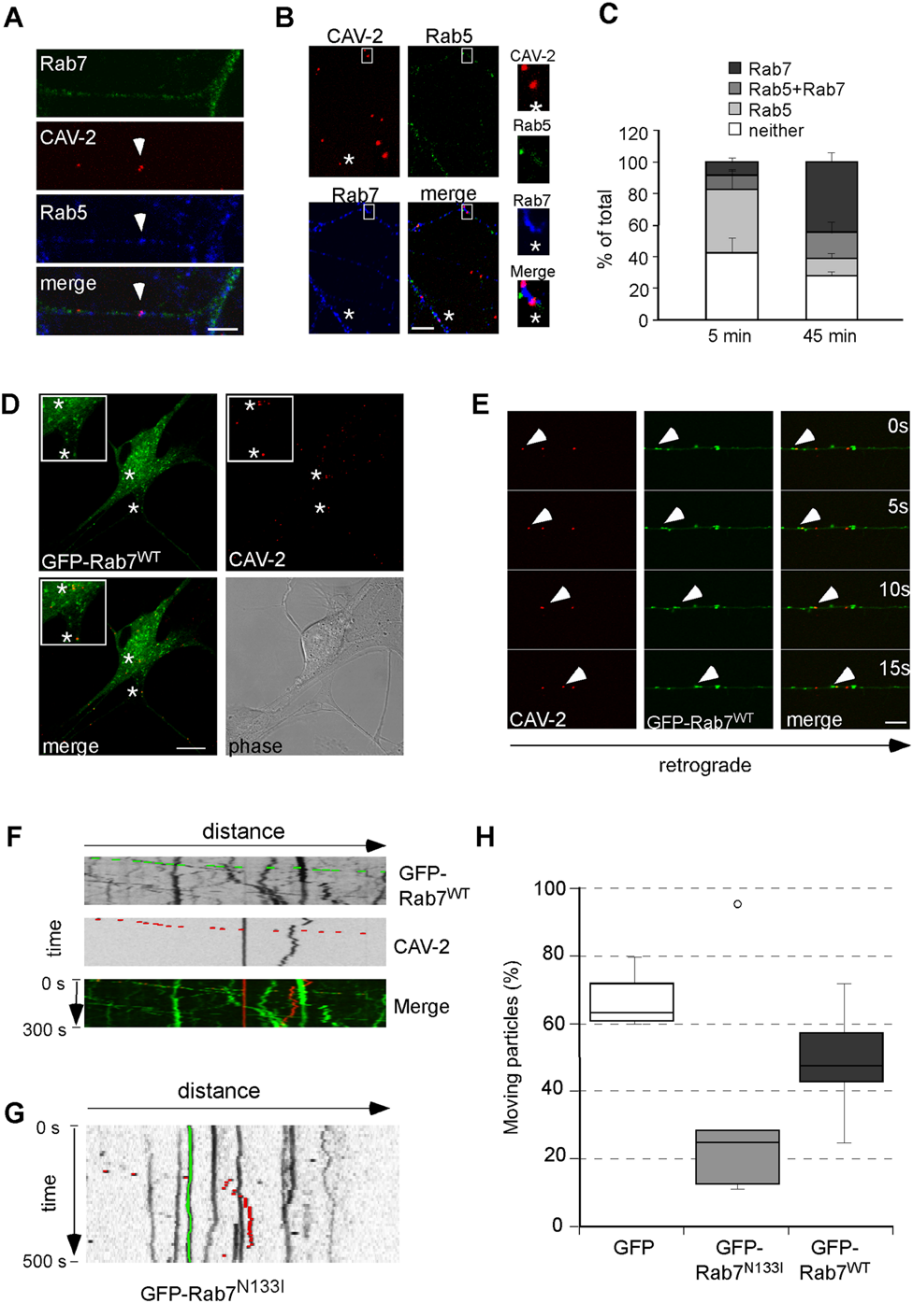
Endosomal maturation during internalisation and transport of CAV-2. (A) MNs were incubated with CAV-Cy3 for 5 minutes at 37°C and stained for endogenous Rab5 and Rab7. The arrow shows CAV-2 present in a Rab5-positive organelle. (B) Immunofluorescence experiments show that 45 minutes post-entry, CAV-2 is present mainly in endosomes containing only Rab7 (asterisk). (C) Quantification of 3 independent experiments shows progression along the endocytic pathway during CAV-2 entry and transport (5 minutes, 141 virions; error bars represent 9% (neither), 11.3% (Rab5), 3.8% (Rab5+7) and 3% (Rab7); 45 minutes, 171 virions; error bars represent 3.1% (neither), 3.1% (Rab5), 6.9% (Rab5+7) and 5.9% (Rab7)). (D) and (E) MNs were microinjected with an expression plasmid encoding GFP-Rab7^WT^ and imaged live after CAV-2 infection. (D) GFP-Rab7^WT^ is localised on a vesicular compartment distributed in the soma and neurites. CAV-2 is associated with GFP-Rab7^WT^-positive organelles in cell bodies and neurites (asterisks). (E) Live cell experiment showing CAV-2 transport in an axonal GFP-Rab7^WT^-positive carrier (arrowhead). (F) Corresponding kymograph. (G) MNs expressing the dominant-negative GFP-Rab7^N133I^ mutant were imaged live after CAV-2 infection. Representative kymograph shows strong reduction of CAV-2 transport. An example of a CAV-2 carrier resuming bidirectional transport after a long pause is highlighted in red, whilst a stopped virion is in green. (H) Quantification of the effects of GFP, GFP-Rab7^WT^ and GFP-Rab7^N133I^ expression on CAV-2 transport. At least 3 independent experiments were performed for each condition (112 (GFP), 107 (GFP-Rab7^WT^) and 45 (GFP-Rab7^N133I^) virions in total. Scale bars: (A, B, E) 5 µm; (D) 10 µm.

Progression along the endocytic pathway is tightly regulated in time and space. In many cell types, the classical endosomal pathway involves early endosomes containing Rab5, which then mature into late endosomes characterised by the presence of Rab7 on their cytosolic face [46]. Because axonal transport of TeNT H_C_ requires the sequential activities of Rab5 and Rab7 [44], we asked if these small GTPases were also associated with CAV-2 transport. MNs were incubated with CAV-Cy3 for 5 or 45 minutes, fixed and stained for endogenous Rab5 and Rab7. At 5 minutes post-internalisation, we found numerous Rab5/CAV-2 structures lacking Rab7, both in axons (Figure 4A) and in cell bodies (data not shown), demonstrating that the virions associated with early Rab5^+^ endosomes immediately after internalisation. However, at 45 minutes post-internalisation we detected virions mainly in Rab7^+^ structures (Figure 4B). Quantitative analysis of the distribution of Rab5, Rab7 and CAV-2 showed that at 5 minutes post-internalisation, 40% of CAV-2 was in Rab5^+^ compartments whereas at 45 minutes post-internalisation, only 11% of the virions colocalised with Rab5. In contrast, at 45 minutes 44% of virions colocalised with Rab7, and 16% were Rab5/Rab7 double positive (Figure 4C). These ratios are in good agreement with the colocalisation between transported TeNT H_C_ and Rab7 [44].

To address the functional relationship between CAV-2 transport and Rab7 activity, we microinjected MNs with plasmids expressing GFP-tagged fusion proteins of either wild-type Rab7 (GFP-Rab7^WT^) or its dominant-negative N133I mutant (GFP-Rab7^N133I^) [47]. The axonal transport of CAV-2 was then assayed using live-cell imaging in GFP and GFP-Rab7 expressing neurons. In agreement with the degree of colocalisation observed with the endogenous protein, CAV-Cy3 colocalised with GFP-Rab7^WT^ in somas (Figure 4D) and axons (32%; 5 independent experiments, 107 virions in total) (Figure 4E and F). Furthermore, the GTPase activity of Rab7 was essential for axonal transport of CAV-2 since overexpression of GFP-Rab7^N133I^ strongly impaired CAV-2 movement (Figure 4G and H), compared to overexpression of GFP or GFP-Rab7^WT^ (Figure 4G and H). In agreement with previous reports [48], the inhibitory effect of GFP-Rab7^N133I^ is linked to its expression levels. As a consequence, sub-threshold GFP-Rab7^N133I^ expression did not alter the axonal transport of CAV-2 (Figure 4H; outlier in the GFP-Rab7^N133I^ sample). Conversely, strong overexpression of GFP-Rab7^WT^ caused a partial, yet not significant, inhibition of this process (Figure 4H). These results suggest that Rab5 to Rab7 vesicular maturation is required for CAV-2 progression along the axonal endocytic pathway.

### The axonal transport of CAV-2 relies on kinesin-1 and cytoplasmic dynein motor activities

**Figure 5:**
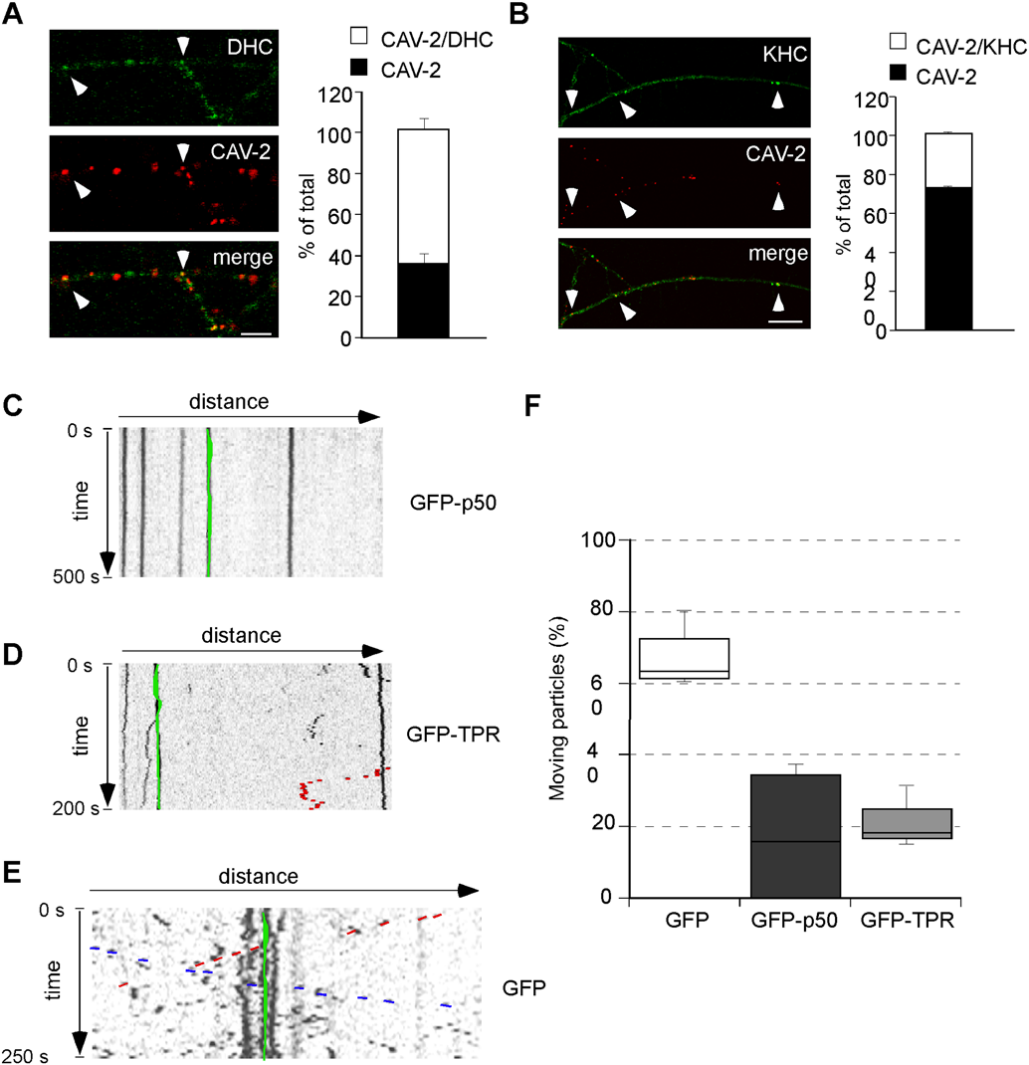
Cytoplasmic dynein and kinesin-1 drive axonal CAV-2 transport in MNs. MNs were incubated for 45 minutes with CAV-Cy3, fixed and stained for molecular motor components. (A) Dynein heavy chain (DHC) colocalises with CAV-2 in MN axons (arrowheads show double-positive structures). Quantification is shown on the right (3 independent experiments, 168 virions in total; error bars represent 5.2%). (B) Kinesin heavy chain (KHC) is associated with a subpopulation of CAV-2 (arrowheads). Quantification of 3 independent experiments is shown on the right (83 virions in total; error bars represent 1.14%). (C–F) MNs were microinjected with either GFP or GFP-p50 or GFP-TPR expression plasmids and imaged live after CAV-Cy3 infection. Representative kymographs show a strong inhibition of CAV-2 transport in either GFP-p50-expressing (C) or GFP-TPR-expressing (D) MNs. An example of a CAV-2 carrier resuming bidirectional transport after a pause is highlighted in red, whilst stopped virions are in green. (E) Kymograph of a GFP expressing neuron showing normal CAV-2 transport. (F) Quantification of the effect of inhibiting cytoplasmic dynein and kinesin 1 on CAV-2 axonal transport. A minimum of 3 independent experiments was performed for each condition; 112 (GFP), 54 (p50) and 52 (TRP) virions in total. Scale bars: (A) 5 µm; (B) 10 µm.

Axonal transport is mainly powered by the microtubule-dependent motors cytoplasmic dynein and kinesins [34]. To further understand the determinants of bidirectional CAV-2 transport, we stained MNs previously incubated in the presence of CAV-Cy3 with antibodies specific for subunits of motor complexes. Dynein heavy chain (Figure 5A) and p50/dynamitin, a subunit of the dynein-dynactin complex (data not shown), were associated with more than 60% of virions, suggesting that this ubiquitous retrograde motor plays a major role in the axonal transport of CAV-2. Secondly, we found a lower, albeit significant, colocalisation of virions with the heavy chain of kinesin-1 (KHC) (Figure 5B). Although these data do not exclude the possibility that the bidirectional transport of CAV-2 is due uniquely to dynein, they favour the likelihood that both cytoplasmic dynein and kinesin play a role in this process. To directly demonstrate the involvement of these motor proteins in CAV-2 transport, we overexpressed p50/dynamitin, a treatment that disrupts endogenous dynein-dynactin complex [49]. In p50/dynamitin-expressing MNs, CAV-2 transport was strongly inhibited (Figure 5C and F) compared to GFP-expressing cells (Figure 5E). Similarly, overexpression of the tetratricopeptide (TPR) domain of kinesin light chain 1 [50] also reduced the frequency of motile virions (Figure 5D and F), suggesting that the axonal transport of CAV-2 require coordination between plus and minus-end microtubule motors.

### CAV-2 and CAR colocalise on axonal carriers

**Figure 6:**
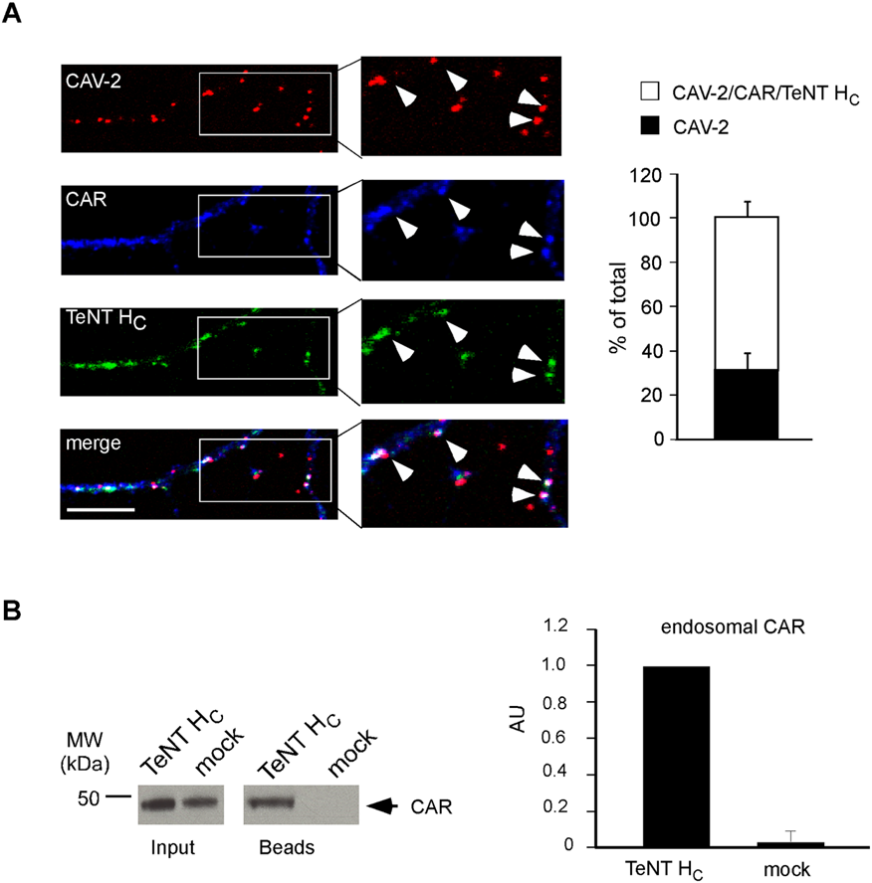
CAV-2 and CAR colocalise in axonal endosomes. (A) CAR colocalises with CAV-2 and TeNT H_C_ in MNs. Acid-washed MNs incubated with CAV-Cy3 and AlexaFluor488-TeNT H_C_ for 45 minutes show internal virions in compartments containing CAR and TeNT H_C_ (arrowheads) in MN axons. Quantification is shown on the right panel (3 independent experiments, 95 carriers in total; error bars represent 7.3%). (B) Magnetic pull-down of TeNT H_C_-containing vesicles. MNs were incubated with TeNT H_C_ coupled to superparamagnetic Fe beads for 60 minutes and then lysed in absence of detergent (see Materials and Methods). A representative experiment (left panel) and its quantification (right panel, 3 independent experiments) are shown. Western blot analysis after magnetic purification shows a specific association of CAR with TeNT H_C_ transport carriers. Scale bars = 10 µm.

Although the binding of Ads to CAR may induce downstream signalling [51], CAR's role in Ad infection has been considered primarily as a docking site prior to integrin-mediated internalisation. Consistent with this, deletion of CAR cytoplasmic tail had no significant effect on Ad internalisation in epithelial cells [52]. Yet, CAV-2 is one of a handful exceptions in the *Adenoviridae* family: the external capsid, in particular the penton base, does not contain a recognisable integrin-interacting motif [16],[53],[54]. Therefore, we asked whether CAV-2 and CAR were associated during endocytosis and the subsequent axonal transport. As mentioned previously, CAR staining in MNs showed a plasma membrane as well as an intracellular localisation (Figure S1A). After 45 minutes post-internalisation, 80% of axonal CAV-2 was found in CAR^+^ structures (Figure S3). Furthermore, upon incubation of MNs with TeNT H_C_ and CAV-2, followed by an acid wash to remove extracellular-bound ligands whilst preserving internalised probes [44], anti-CAR immunostaining revealed high colocalisation levels of CAR, CAV-2 and TeNT H_C_ in neurites (∼70%; Figure 6A). The colocalisation of CAV-2 and TeNT H_C_ in axonal carriers prompted us to use a biochemical approach based on TeNT H_C_-coupled to superparamagnetic nanobeads to isolate these transport vesicles [44]. Using western blot analysis, we detected an ∼250-fold enrichment of CAR in these organelles (Figure 6B), further supporting the notion that CAR and CAV-2 co-inhabit a pool of axonal transport vesicles.

### CAR endocytosis is linked to the axonal transport machinery

**Figure 7:**
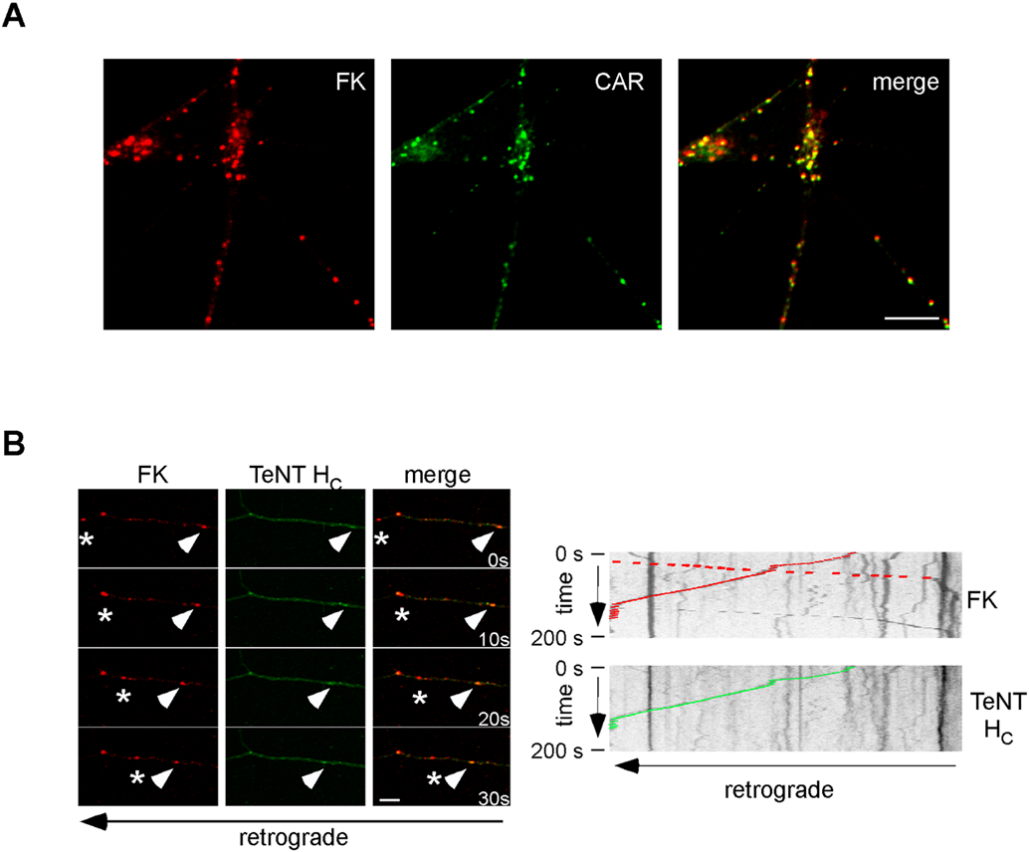
CAR internalisation and bidirectional transport in neurons. (A) MNs were incubated with FK-Cy5 (pseudo-coloured in red) for 45 minutes, acid washed, fixed and stained for endogenous CAR. FK and CAR colocalise in a vesicular compartment in the cell body and axons of MNs (>95%, 3 independent experiments, 412 FK structures in total). (B) MNs were incubated with FK-Cy5 (pseudo-coloured in red) and AlexaFluor488-TeNT H_C_ for 45 minutes and imaged. Still images of a movie are showed. Arrowhead shows a retrograde carrier containing both FK and TeNT H_C_; the asterisk shows an anterograde organelle containing FK. A kymograph showing the bidirectional transport of FK in the right part of panel B. Scale bars: (A) 10 µm; (B) 5 µm.

To directly monitor CAR neuronal trafficking, we used fluorescently-labelled CAV-2 fibre knobs (FK-Cy5 and FK-Cy3) to visualise CAR entry and transport in MNs. Initially, we tested the specificity of labelled-FK binding to CAR by transfecting CAR-negative NIH 3T3 cells with a plasmid encoding a GFP-CAR fusion protein. Transfected cells were then incubated with FK-Cy5 and fixed. We found that only GFP^+^ cells bound FK-Cy5, strongly supporting a CAR-specific binding of the CAV-2 fibre knob FK-Cy5 (Figure S4A). Consistently, preincubation of MNs with unlabelled FK blocked FK-Cy5 labelling (Figure S4C). When MNs were incubated with FK-Cy5 followed by acid wash, FK and CAR colocalised in discrete puncta (>95%, Figure 7A), suggesting that this viral protein and its cellular receptor are linked during endocytosis. Furthermore, FK-Cy5 was retrogradely transported in the same carriers as TeNT H_C_ and displayed a bidirectional transport similar to CAV-2 (Figure 7B), suggesting that CAR-mediated binding and internalisation is coupled to axonal transport. Accordingly, we also found FK-Cy5 in GFP-Rab7^+^ axonal carriers (data not shown).

To further understand the role of CAR in CAV-2 binding and endocytosis, we took advantage of a CAR-ablated FK variant (FKm), which bears a single-point mutation in the CAR binding site [15]. We incubated MNs on ice with labelled-FK or FKm. In these conditions, FKm was not able to bind MNs (Figure S4B). Together, these results strongly suggest that in neurons, CAR can be endocytosed and trafficked bidirectionally in axons, and that this protein may dictate internalisation and subsequent axonal transport of CAV-2.

### CAR is transported in axons of the sciatic nerve *in vivo*

**Figure 8:**
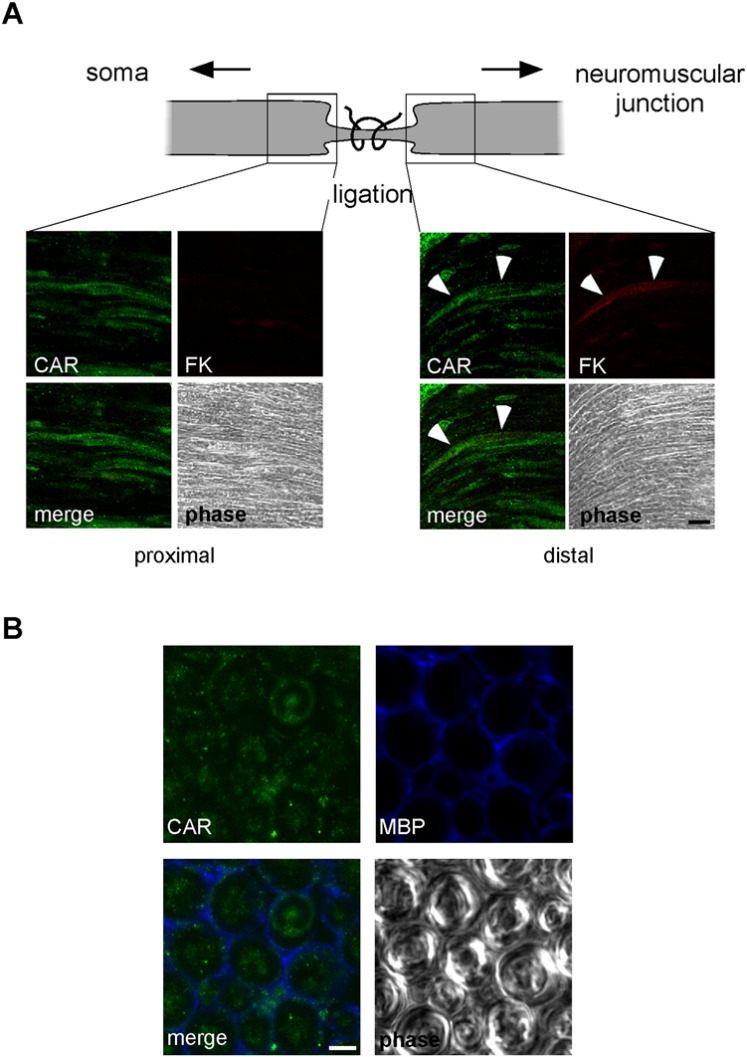
CAR transport in sciatic nerve axons. (A) To monitor axonal transport of CAR *in vivo*, FK-Cy3 was injected into the tibialis anterior and gastrocnemius muscles after ligation of the sciatic nerve. A representative experiment is shown. FK was only found in axons in the distal part of the ligature (right panel; see also the scheme of the experiment on the top part of the figure). These axons are positive for CAR (right panel; in green; arrows). CAR accumulates also at the proximal side, suggesting that it undergoes bidirectional transport *in vivo*. FK was undetectable on the proximal side of the ligation (left panel; in red). (B) Transverse section of an unligated sciatic nerve shows intra-axonal CAR. Myelin basic protein (MBP) delimits axons. Scale bars: (A) 10 µm; (B) 5 µm.

The above results suggest that Ads take advantage of an innate trafficking of CAR to access the CNS. This prompted us to investigate its intracellular dynamics *in vivo*. Sciatic nerve ligation represents a powerful system to study axonal dynamics. To specifically monitor CAR axonal transport, we injected FK-Cy3 in the tibialis anterior and gastrocnemius muscles of C57BL/6 mice after ligation of the sciatic nerve. Eight hours post-injection, we examined the distributions of CAR and FK-Cy3. Consistent with our hypothesis, CAR accumulated inside axons in both proximal and distal parts of the ligation site (Figure 8A). However, only distal sections showed a clear signal correspondent to retrogradely-transported FK-Cy3 (Figure 8A, right panel). Intra-axonal CAR was also observed by staining for CAR in transverse sections of unligated sciatic nerve (Figure 8B). CAR distribution was not significantly affected by the presence of FK-Cy3 since similar CAR staining patterns were also observed in the absence of FK (Figure 8A left panel, B, and data not shown). These data suggests that CAR undergoes constitutive bidirectional transport in sciatic nerve *in situ*.

## Discussion

A better understanding of the interactions between adenovirus and neurons was essential and overdue. To our knowledge, this is the first study to address the determinants of Ad neurotropism and axonal transport. Axonal transport has been described for a handful of viruses, including rabies, herpes simplex type I (HSV-1), measles, West Nile and poliovirus. Although less common than the above pathogens, both human and canine Ad serotypes are associated with brain pathologies [3],[4]. Notably different mechanisms of axonal transport have been described: direct interaction with molecular motors for HSV-1 and rabies viruses [55] versus endosomal trafficking for poliovirus [56].

Our proposed model goes partially against the paradigm derived from prototype Ad trafficking studies performed in epithelial cells. We propose that the recognition of CAV-2 on the neuronal surface is primarily CAR-dependent. Internalisation involves CAR and clathrin-coated vesicles that acquire the early endosomal marker Rab5, yet apparently does not induce capsid disassembly and endosomal escape. These latter axonal vesicles mature into Rab7^+^ compartments that still contain CAR, and have the advantageous characteristic of being nonacidic. After a lag phase, long-range transport of CAV-2 entrapped in vesicular organelles becomes sustained and bidirectional, probably involving the concerted action of dynein and kinesin. Crucially, our data also suggest an innate function of CAR in axons dictating CAV-2 transport.

Endocytic progression is required for Ad infection and has been shown to differ mechanistically for different Ad serotypes [28]. The lag phase observed before the onset of CAV-2 axonal transport, which is not seen in epithelial cells infected by CAV-2 or Ad2/5 [30],[39], was also similar to that observed for TeNT H_C_ and p75^NTR^ [44]. Although further studies will be needed to pinpoint the underlying causes of this delayed onset, a likely explanation is that it is due to cargo sorting and/or endosome maturation. The association of CAV-2 initially with Rab5^+^ early endosomes and then with a transport compartment containing Rab7 is also similar to TeNT H_C_ trafficking. Interestingly, Rab7 effectors RILP and ORP1L can mediate the recruitment of cytoplasmic dynein to endosomes in HeLa cells [57]. Whether Rab7 also directly recruits the dynein complex in axons is unknown, but might explain why, by reaching organelles containing Rab7, CAV-2 undergoes efficient axonal transport. Although other serotypes can reach Rab7^+^ compartments [45], there appears to be a functional difference between some of those found in axons and epithelial cells, one difference being that a population of Rab7^+^ endosomes in axons have lumens that are neutral. Using a marker described to traffic inside pH-neutral carriers (TeNT H_C_), CAV-2 linked to a pH-sensitive dye [43] and Lysotracker, we showed that in contrast to virions in the cell body that can reach acidic organelles (pH 5–5.5), the majority of axonal CAV-2 carriers had a pH ranging from 6 to 7. These data, combined with previous report of the pH of axonal organelles [58] demonstrate that the presence of Rab5 and Rab7 offer no indication of the pH of the endosomes or other organelles under investigation. Neurons appear to differ in the regulation of endosomal acidification that occur in their axons versus cell body. By entering nonacidic organelles in axons, CAV-2 could remain stably and efficiently associated with long-range carriers until delivered to the soma, where endosomal acidification could occur, triggering the exit from these compartments.

In light of these results, it is tempting to speculate that human Ad serotype 5 (HAd5), which can be retrogradely transported *in vivo* [31],[32] and escapes endosomes when the pH drops below 6 in epithelial-like cells [45], could take advantage of a similar protective endocytic pathway to reach the neuronal cell body. Interestingly, when HAd5 and CAV-2 vectors were mixed and co-injected in the rodent brain, both are capable of axonal transport to afferent regions. However CAV-2 vectors are 50–100 fold more efficient when transgene expression is used as a readout at distal sites [7]. Does HAd5 use a pathway similar to CAV-2? There are notable similarities and differences between HAd5 and CAV-2 that may affect their axonal transport. In the case of CAR as a binding site, our data have consistently suggested that CAV-2 is “CAR-tropic” while other studies have reported that HAd5 uses CAR, as well as other cell surface molecules for binding and internalisation [37]. CAV-2 is also more thermostable than HAd5 (unpublished data). *A priori*, we would predict that if an HAd5 virion binds CAR it could be taken up and transported in a manner similar to that seen by CAV-2. Using real time confocal microscopy we detected fast axonal transport of HAd5 in primary neurons (our unpublished data) suggesting, but not demonstrating, similarities in transport. We do not know if the increased thermal stability of CAV-2 versus HAd5 plays a role during vesicular maturation at, for example, the axon soma interface. The interaction with integrins via the HAd5 penton base may also make the HAd5 capsid more sensitive to disassembly triggers in the lumen of a Rab7 vesicle in axons.

The motility of CAV-2 showed an average retrograde speed above 1 µm/s, consistent with fast axonal transport. Notably, we found a minor population of CAV-2 and FK carriers undergoing bidirectional transport. Similar bidirectional transport was detected using FK to monitor CAR trafficking in axons. This feature is not unique to Ad: HSV-1 shows bidirectional transport with a bias for the retrograde direction during infection and displays a preferential anterograde transport during the phase of egress [55],[59]. However, bidirectional HSV-1 transport is via direct recruitment of motors to the viral capsid. The association of CAV-2 and CAR with organelles undergoing bidirectional movement is particularly interesting because the regulation of bidirectional transport is still poorly understood. In this regard, CAR- or CAV-2-containing endosomes could represent an ideal tool to address how vesicular cargo coordinates the recruitment of both classes of microtubule-dependent molecular motors, or how a main retrograde motor, such as cytoplasmic dynein, may switch to an anterograde direction [40]. Dynactin may be a potential regulator of kinesin- and dynein-driven transport since it is able to simultaneously bind these two classes of motors. Interestingly, p50/dynamitin, a subunit of the dynactin complex, colocalised with CAV-2, and p50/dynamitin overexpression inhibited the axonal transport of virions. The observed impairment of CAV-2 transport by inhibition of either cytoplasmic dynein or kinesin-1 suggests that coordination between these two classes of motors is necessary to ensure efficient axonal retrograde transport, as previously observed for TeNT H_C_ carriers and mitochondria (reviewed in [33]).

*A priori*, one could envisage that the internalised cargo, via its interaction with specific integral membrane proteins, dictates the directionality of the transport. In this light, although TeNT H_C_ and CAV-2 share a high number of axonal carriers, together they move exclusively in the retrograde direction. In contrast, anterograde moving organelles contain CAR and CAV-2, but lack TeNT H_C_. This observation suggests the existence of discrete sorting steps during internalisation or *en route* endosomal maturation, which alter the ability of transported endosomes to recruit or activate anterograde and/or retrograde motor complexes. This may be achieved by engaging specific adaptor proteins able to co-ordinate motor complex activity, as in the case of huntingtin, which controls the directionality of vesicular carriers in cortical neurons via an Akt-dependent phosphorylation switch [60].

Although CAR is the main receptor for many Ad serotypes, little is known regarding its intracellular dynamics in neurons. In addition to a plasma membrane targeting, we found that CAR is also present on an internal vesicular pool. By means of competition experiments, we showed that the binding to CAR is an essential step for the entry of CAV-2. CAV-2 and its recombinant FK are taken up in CAR-containing vesicles, suggesting that the virus and its receptor could be endocytosed together and then co-transported. Notably though, our assays do not address whether fibres detach from the capsid, which is an early step in virion disassembly in epithelial cells [28]. Given the average size of CAV-2^+^ vesicles (100–110 nm) versus the CAV-2 icosahedra core (∼90 nm [61] plus the projecting fibres (30 nm)), the most obvious prediction is that the fibres would be detached. However, the CAV-2 fibre shaft, in contrast to other Ads [36], is particularly flexible due to the presence of two hinges [61]. This added suppleness may allow the fibre to fold over whilst still attached to CAR in the lumen of the endosomes.

By using fluorescently-labelled CAV-2 FK, we also demonstrated that CAR undergoes endocytosis and bidirectional transport in cultured MNs and in sciatic nerve axons. These findings introduce a paradigm shift for the CAR-mediated endocytosis of Ads. As mentioned above, the available *in vitro* evidence is that CAR functions as a primary attachment site and that integrins are responsible for virus internalisation via the interaction with motifs in the Ad penton base. The homotrimeric FK could bind three CAR D1 domains simultaneously [13],[15],[62]. In this light, it will be critical to determine if the FK induces clustering of CAR, which in turn triggers internalisation of ligand-receptor clusters, or if other mechanisms are involved. Interestingly, the affinity of the CAV-2 FK to CAR is 5 to 7-fold times greater than that of HAd5 knob-CAR and the highest reported for an Ad [15].

The roles of CAR as an adhesion molecule and key component of tight junctions are well established [18]. Although CAR is highly expressed in the developing brain [17], its neuronal function(s) remains speculative. Based on its direct interaction with actin, a potential role of CAR in neurite outgrowth has been proposed [63]. Recently, this association has been extended to several cytoskeletal components, suggesting a more general role of CAR in actin and microtubule dynamics [63],[64]. Notably, our *ex vivo* and *in vivo* data demonstrate that CAR is found inside axons even in absence of an exogenous “ligand”, and also link CAR directly or indirectly to the axonal transport machinery. Together, our observations suggest that CAV-2 is taking advantage of an axonal trafficking pathway involving CAR and that allows virions to be efficiently transported to the CNS.

The nature and regulation of axonal transport pathways are of crucial interest since their impairment has been linked to several neurodegenerative disorders. In this context, some Rab7-associated axonal organelles may be the hallmark of a long-range, vectorial axonal transport. Because CAV-2, like TeNT, is able to reach this compartment, it may have a preferential and efficient access to the CNS. Indeed, this Rab7^+^ nonacidic axonal compartment may offer ultimate protection against degradation during long-range transport, allowing pathogens, virulence factors, as well as endogenous molecules, to be delivered intact to the cell body of neurons.

## Materials and Methods

### Ethics Statement

All experiments were carried out under license from the UK Home Office in accordance with the Animals (Scientific Procedures) Act 1986 and following approval from the Cancer Research UK Ethical Review Committee.

### Materials and neuronal cultures

Labelling reagents, AlexaFluor488-Lysotracker, AlexaFluor647-dextran, carboxyfluorescein and AlexaFluor-conjugated secondary antibodies were from Invitrogen. Mouse monoclonal anti-CAR antibody (MoAb.E(mh); a gift from Steven Carson, University of Nebraska) was used at 1∶500 in western blot analyses. Rabbit polyclonal anti-CAR antibodies (1∶300) (Ab1605; a gift from Joseph Zabner, University of Iowa), monoclonal anti-Rab5 (1∶200; Synaptic System), polyclonal anti-Rab7 (1∶200) [44], polyclonal anti-FK (1∶300) [65], anti-DHC (1∶100; Santa-Cruz) anti-p50/dynamitin (1∶200; BD Bioscience), anti-KHC (1∶100; Chemicon) were used in immunofluorescence (IF) studies. Monoclonal anti-Cy3 (1∶200; Abcam) was used on live cells. Anti-MBP was purchased from Boehringer (Mannheim, Germany). p50/dynamitin and TPR construct were kindly provided by Michael Way (CRUK, London). The plasmid expressing GFP and CAR was a gift from Joseph Zabner. Paramagnetic Fe-beads were purchased from G. Kisker GbR. Rat spinal cord MNs were purified from E13.5 embryos as described previously [43] and used from day *in vitro* 5 onwards.

### Vectors and viruses

CAV-Cy3 was prepared from the E1-deleted vector CAVGFP [66] by direct post-purification labelling with Cy3 [39]. CAV-Cy3 has a physical particle (pp) to infectious unit (IU) ratio of 25∶1 [66]. The vector was propagated, purified, and titred as previously described [7],[66]. Multiplicities of infection are in pp/cell.

### Immunofluorescence, live cell imaging and data quantification

For internalisation assays, MNs were incubated with CAV-Cy3 on ice and either fixed or shifted to 37°C for 45 minutes, back on ice, incubated with anti-Cy3 to label cell-surface virions and then fixed. Indirect immunofluorescence (IF) experiments were performed as follow. After fixation, MNs were permeabilised with 0.1% Triton X-100 for 5 minutes at room temperature (RT), followed by blocking with 3% bovine serum albumin (BSA) for 1 hour at RT. Primary and secondary antibodies were diluted in blocking solution and incubated sequentially for 1 hour at RT. Samples were then mounted with Mowiol (Harco) and imaged by confocal microscopy. For live cell experiments, MNs were incubated with CAV-Cy3 and AlexaFluor488-TeNT H_C_ or AlexaFluor647-dextran or AlexaFluor488-Lysotracker at 37°C, washed with DMEM containing 30 mM HEPES-NaOH, pH 7.4 and imaged. Live and fixed samples were imaged by confocal microscopy (Zeiss LSM 510 equipped with a 63×, 1.4 NA Plan Apochromat oil-immersion objective). Images were processed using Zeiss LSM 510 software. For live cell imaging, 100–150 frames were acquired (0.2 frames/s) and analysed as previously described [42]. Kymographs were generated using MetaMorph (Molecular Devices). Vertical single line-scans through the thickness of each process were plotted sequentially for every frame in the time series. Acid wash was performed to release proteins bound to the cell surface by incubating the cells for 5 minutes at room temperature in 100 mM citrate-NaOH, pH 2.0, 140 mM NaCl and washed with PBS. Virus binding was quantified using the spot count option of the Imaris software and normalized to the total amount of membrane measured by voxel counting using ImageJ.

### Intracellular pH measurement

CAV-2 was directly labelled with carboxyfluorescein according to a previous report [45]. Briefly, carboxyfluorescein can be used as intracellular pH sensor by measuring the ratio of emission intensities upon sequential excitation at 458 and 488 nm (I^488^/I^458^). CAV-CF-infected MNs were imaged live and after obtaining the calibration curve (with MNs treated with 10 µg/ml of nigericin and monensin+L15 at various pHs), axonal versus somatic particles emission intensities were analysed. Intensities and ratios were measured using imageJ (version 1.37).

### Magnetic isolation of axonal retrograde carriers and image quantification

Magnetic isolation of TeNT H_C_ carriers was performed as previously described [44]. Quantification of CAR enrichment in carriers by western blot was performed using ImageJ.

### Recombinant proteins and fluorescent labelling

TeNT H_C_ was isolated and labelled as previously described [44]. CAV-2 FK (residues 358–542) construct was cloned into pPROEX HTb (Life Technologies), expressed with a cleavable His_6_-tag, and purified as previously described [15]. The CAV-2 FKs were dialysed in PBS 0.1 M Na_2_CO_3_ pH 9.3 and labelled using Cy5 mono-reactive dye pack (Amersham Bioscience) for 45 minutes at RT. The elution of labelled protein was performed with 2 ml of PBS using NAP5 column (GE Healthcare) pre-equilibrated with 10 ml PBS. The final dye/protein ratio (∼2.4) was determined using a NanoDrop ND-100 spectrophotometer.

### Transmission electron microscopy (TEM)

For TEM analysis, MNs were incubated for various time periods with CAV-Cy3. Cells were washed twice with 0.2 M Sorensen's buffer and fixed with 2.5% glutaraldehyde (Agar) in Sorensen's buffer, containing 70 mM sucrose for 1 h at 4°C. After washing, MNs were post-fixed with 1% osmium tetroxide for 30 minutes, washed, dehydrated in an ascending ethanol series and embedded in araldite over 2 days. Thin sections were stained with methanolic uranyl acetate and lead citrate. Sections were imaged with a JEOL 1010 transmission electron microscope.

### Sciatic nerve ligation and intramuscular injection of FK-Cy5

Under isoflurane anaesthesia (National Veterinary Services, Stoke on Trent, UK), an incision was made along the left flank of adult C57Bl/6 mice to expose their sciatic nerve, which was ligated at the mid-thigh level. Immediately following ligation, the tibialis anterior and gastrocnemius muscles were exposed and FK-Cy5 (6 µg in 8 µl) was slowly injected intramuscularly using a Hamilton microsyringe. The needle was left in place for 1 minute to prevent leakage. The wound was sutured and the animals were allowed to recover. After approximately 8 hours, the mice were terminally anaesthetized with sodium pentobarbitone and perfused transcardially with 4% PFA (TAAB) in 0.1 M PBS. The ligated sciatic nerve was removed, post-fixed for 4 hours in the same fixative and then cryoprotected in 30% sucrose in PBS. The animals were housed in a controlled temperature and humidity environment and maintained on a 12 hour light/dark cycle with access to food and water *ad libitum*.

## Supporting Information

Figure S1 CAR neuronal localisation and CAV-2 entry. (A). Primary MNs in culture were fixed, permabilised and fixed for endogenous CAR. a- Confocal analyses showed internal structures containing CAR in MN. Left panel shows z-stacks of a MN. Asterisks highlight internal CAR. b and c- Epithelial-like cells in the culture displays only cell-to-cell contact localisation of CAR. (B) MNs were incubated on ice with CAV-Cy3, fixed and stained by indirect immunofluorescence for CAR. Axons display some punctate staining of CAR that colocalise with CAV-2. (C) Cells treated with CAV-2 for 1 minute were fixed and imaged by TEM. White arrow shows a structure ressembling a clathrin-coated pit. (D) MN were incubated for 2 min with CAV-2-Cy3, fixed and stained for clathrin heavy chain (CHC). Arrowheads show virions associated with CHC. Scale bar: (A and B) 10 µm (C) 100 nm (D) 5 µm.
(1.75 MB PDF)

Figure S2 Kinetics of CAV-2 retrograde transport. MNs were incubated with CAV-Cy3 and imaged. Kymographs of CAV-2 transport after 15, 25 and 35 min of internalisation show that the onset of transport occurs after an initial lag phase of 25–30 min. Red dots highlight a still carrier (15 min after internalisation) and a long range transported carrier (35 min after internalisation).
(0.11 MB PDF)

Figure S3 CAV-2 is endocytosed with CAR. MNs were incubated CAV-Cy3 for 45 min, fixed and stained for CAR. The majority of CAV-2 was found together with CAR (arrowheads) (>77% 138 particles, 3 independent experiments. Error bar represent 5.5%). Scale bar: 5 µm.
(0.15 MB PDF)

Figure S4 CAV-2 Fibre Knob (FK) recognises specifically CAR. (A) NIH 3T3 cells were transfected wit GFP-CAR and incubated with Cy5-FK. Only CAR-expressing cells were able to bind FK. (B) FK mutated in the CAR binding site (FKm) does not bind MNs. MNs were incubated on ice with FK or FKm, washed and then fixed prior to confocal imaging. Membranes were revealed by wheat germ agglutinin (WGA). (C) FK-Cy5 binds specifically to the MN surface. MNs were incubated with FK-Cy5 with or without pre-incubation with saturating concentration of unlabelled FK. Scale bars: (A, C) 10 µm, (B) 20 µm.
(0.76 MB PDF)

Video S1 Scanning confocal imaging of CAV-2 infected motor neurons.
(5.55 MB AVI)

Video S2 Scanning confocal imaging of CAV-Cy3 and TeNT Hc-Alexa488 in motor neuron axons.
(10.31 MB AVI)
