# Supplementary figures and images for: CAR-Associated Vesicular Transport of an Adenovirus in Motor Neuron Axons
Source: PLoS Pathog. 2009 May 22;5(5):e1000442. doi: 10.1371/journal.ppat.1000442 (PMC2677547; doi:10.1371/journal.ppat.1000442)

**A**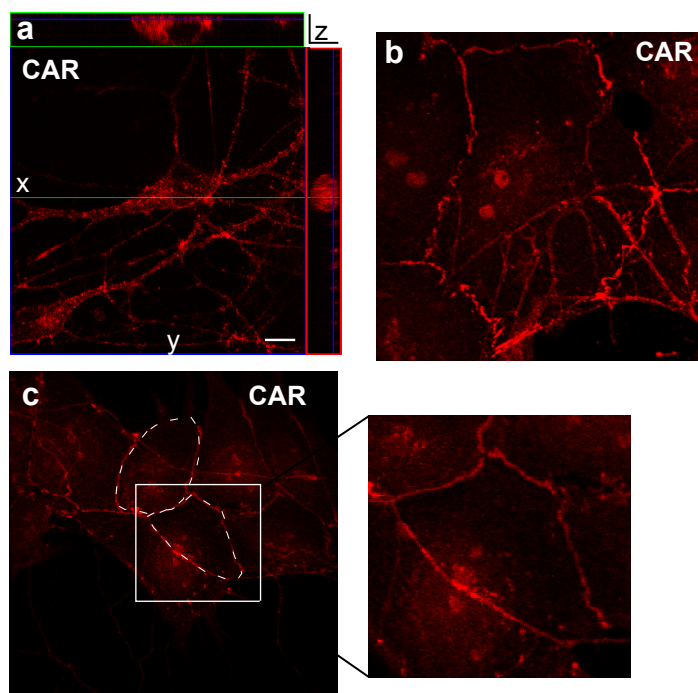**B**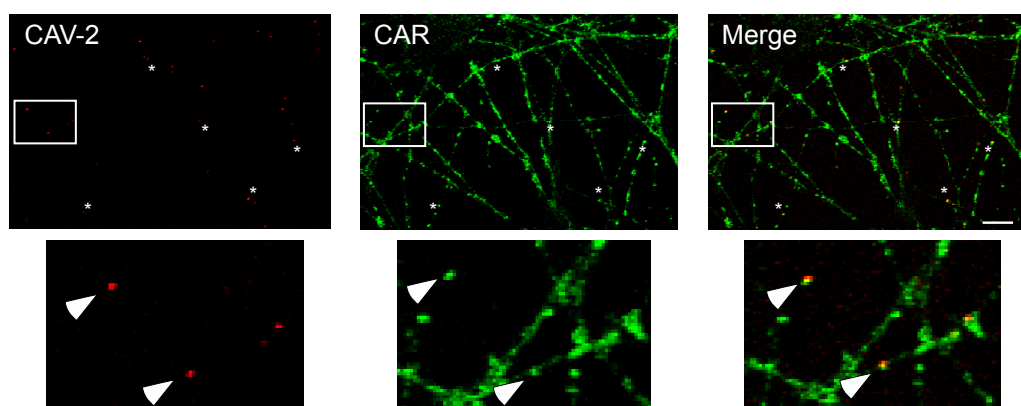**C**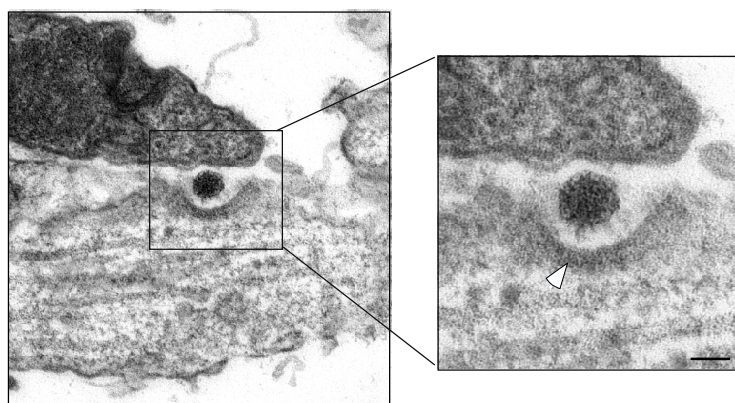**D**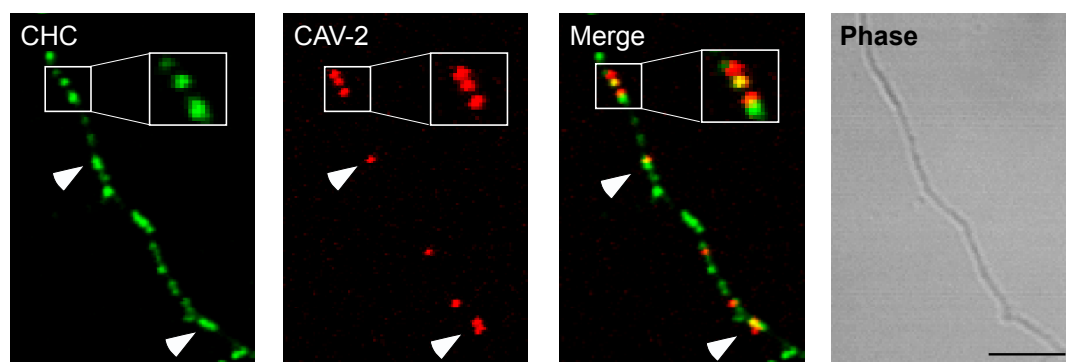

Supplement: Figure S1 — CAR neuronal localisation and CAV-2 entry. (A). Primary MNs in culture were fixed, permabilised and fixed for endogenous CAR. a- Confocal analyses showed internal structures containing CAR in MN. Left panel shows z-stacks of a MN. Asterisks highlight internal CAR. b and c- Epithelial-like cells in the culture displays only cell-to-cell contact localisation of CAR. (B) MNs were incubated on ice with CAV-Cy3, fixed and stained by indirect immunofluorescence for CAR. Axons display some punctate staining of CAR that colocalise with CAV-2. (C) Cells treated with CAV-2 for 1 minute were fixed and imaged by TEM. White arrow shows a structure ressembling a clathrin-coated pit. (D) MN were incubated for 2 min with CAV-2-Cy3, fixed and stained for clathrin heavy chain (CHC). Arrowheads show virions associated with CHC. Scale bar: (A and B) 10 µm (C) 100 nm (D) 5 µm. (1.75 MB PDF) [file ppat.1000442.s001.pdf]

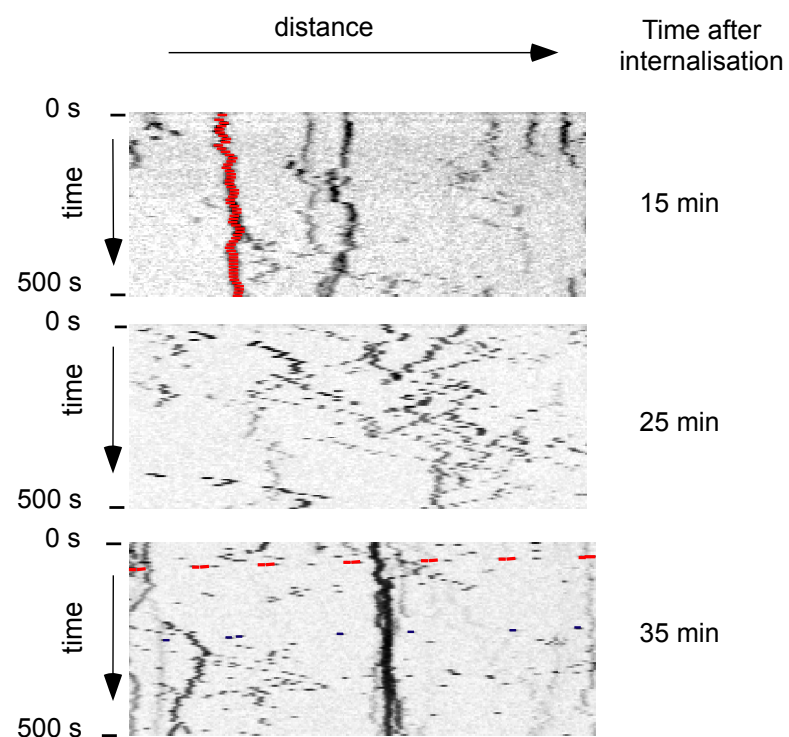

Salinas et al. Figure S2

Supplement: Figure S2 — Kinetics of CAV-2 retrograde transport. MNs were incubated with CAV-Cy3 and imaged. Kymographs of CAV-2 transport after 15, 25 and 35 min of internalisation show that the onset of transport occurs after an initial lag phase of 25–30 min. Red dots highlight a still carrier (15 min after internalisation) and a long range transported carrier (35 min after internalisation). (0.11 MB PDF) [file ppat.1000442.s002.pdf]

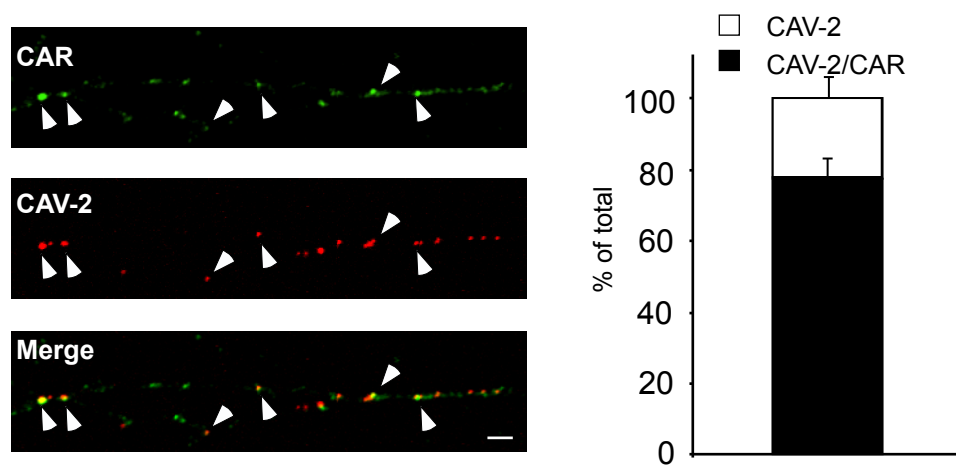

Salinas et al. Figure S3

Supplement: Figure S3 — CAV-2 is endocytosed with CAR. MNs were incubated CAV-Cy3 for 45 min, fixed and stained for CAR. The majority of CAV-2 was found together with CAR (arrowheads) (>77% 138 particles, 3 independent experiments. Error bar represent 5.5%). Scale bar: 5 µm. (0.15 MB PDF) [file ppat.1000442.s003.pdf]

**A**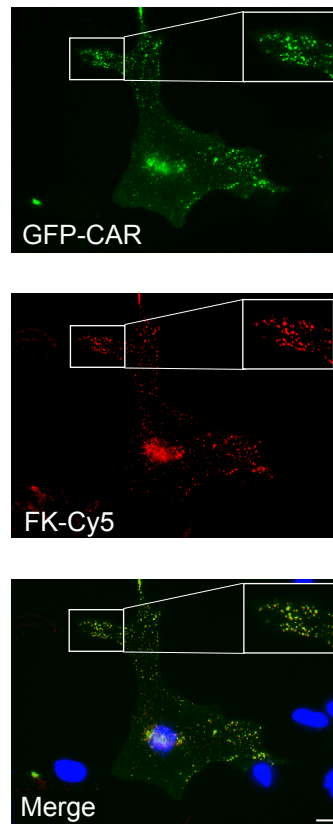**B**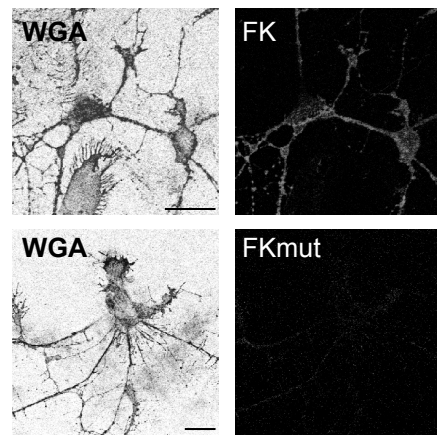**C**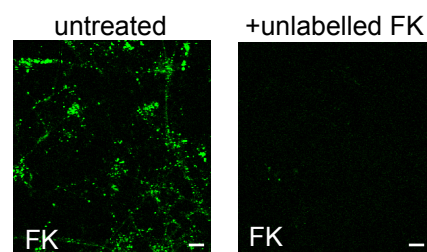

Supplement: Figure S4 — CAV-2 Fibre Knob (FK) recognises specifically CAR. (A) NIH 3T3 cells were transfected wit GFP-CAR and incubated with Cy5-FK. Only CAR-expressing cells were able to bind FK. (B) FK mutated in the CAR binding site (FKm) does not bind MNs. MNs were incubated on ice with FK or FKm, washed and then fixed prior to confocal imaging. Membranes were revealed by wheat germ agglutinin (WGA). (C) FK-Cy5 binds specifically to the MN surface. MNs were incubated with FK-Cy5 with or without pre-incubation with saturating concentration of unlabelled FK. Scale bars: (A, C) 10 µm, (B) 20 µm. (0.76 MB PDF) [file ppat.1000442.s004.pdf]
